# Longitudinal changes in bone mineral density, bone turnover markers, and body composition in children and adolescents treated for acute lymphoblastic leukemia

**DOI:** 10.3389/fendo.2026.1901073

**Published:** 2026-10-19

**Authors:** Alessandro Cattoni, Silvia Molinari, Giulia Capitoli, Silvia Vai, Silvia Carrara, Emanuela Galliera, Luca Massaccesi, Maria Laura Nicolosi, Silvia Radaelli, Martina Sarra, Daniele Tondelli, Rocco Corso, Giovanni Speziali, Andrea Biondi, Adriana Balduzzi, Sabrina Corbetta, Alessandra Sala

**Affiliations:** 1 Department of Pediatrics, Fondazione IRCCS San Gerardo dei Tintori, Monza, Italy; 2 Biostatistics and Clinical Epidemiology, Fondazione IRCCS San Gerardo dei Tintori, Monza, Italy; 3 Bicocca Bioinformatics, Biostatistics and Bioimaging B4 Centre, Department of Medicine and Surgery, University of Milano-Bicocca, Monza, Italy; 4 UOS Bone Metabolic Diseases and Diabetes, IRCCS Istituto Auxologico Italiano, Milan, Italy; 5 Department of Biomedical Sciences for Health, University of Milan, Milan, Italy; 6 IRCCS Ospedale Galeazzi-Sant’Ambrogio, Milan, Italy; 7 School of Medicine and Surgery, University of Milano-Bicocca, Monza, Italy; 8 Department of Radiology, Fondazione IRCCS San Gerardo dei Tintori, Monza, Italy; 9 Department of Biomedical, Surgical and Dental Sciences, University of Milan, Milan, Italy

**Keywords:** acute lymphoblastic leukemia, body composition, bone mineral density, childhood, osteoporosis, sarcopenia

## Abstract

**Purpose:**

Impaired bone mineral density (BMD) is a common finding in young patients with acute lymphoblastic leukemia (ALL). Nevertheless, longitudinal trendlines of BMD and the impact of body composition (BC) on bone health still need to be explored among pediatric survivors. The objective of the study is to report longitudinal changes in BMD and BC in pediatric patients.

**Methods:**

This prospective, longitudinal study, collecting BMD and BC (assessed by dual-energy X-ray absorptiometry [DXA]) and circulating bone turnover markers every 6 months for 3 years, from diagnosis of ALL (T_0_) until 1 year after the end of antiblastic treatment (T_6_).

**Results:**

Forty-nine patients were enrolled. At T_0_, lumbar spine (LS-)BMD Z-score was ≤−2.0 in 71.7% of patients [median: −2.8 (−3.5, −1.9)], possibly reflecting trabecular bone infiltration by lymphoblasts. Conversely, total body less than head (TBLH)-BMD Z-score was >−2.0 in 97.8% of patients at T_0_ but abruptly declined after 6 months (*p* < 0.001). Despite a recovery trend after the end of treatment, LS or TBLH-BMD Z-scores were still <−2.0 in 67.7% of the 31 patients still enrolled at T_6_. Bone turnover was increased between T_1_ and T_4_. Lean mass decreased over time, while fat mass progressively increased. Multivariable analysis showed a statistically significant association between appendicular lean mass longitudinal recovery over time and T_1_–T_6_ TBLH-BMD trendlines (*p* = 0.008).

**Conclusions:**

In pediatric patients, LS-BMD is remarkably affected at diagnosis of ALL. TBLH-BMD, normal at diagnosis, promptly declines during treatment and baseline values are not retrieved after 36 months. Lean mass is positively associated with longitudinal improvements of subtotal BMD over time.

## Introduction

Acute lymphocytic leukemia (ALL) is the most frequent pediatric malignancy, accounting for 28% of tumors in children younger than 14 years and 13% of adolescents aged 15–19 years, with an overall incidence of 58.7 cases per million pediatric population per year (1).

Over the last four decades, the gain of expertise in well-established chemotherapy protocols, a tailored multidisciplinary approach and the development of new targeted treatments has resulted in a dramatic increase in survival rates, currently approaching 90% (1, 2). Accordingly, the management of early and late adverse effects of antineoplastic therapy has become pivotal within the long-term care of children, adolescents and young adults (CAYA) surviving cancer.

Among adverse effects, bone disorders may become overt soon after diagnosis (3), during the first phases of treatment (4), or they may remain latent for many years, until adult age.

Accordingly, the incidence of fractures upon ALL onset and during treatment protocols ranges from 16% to 36% in different series, occurring mainly within the first 2 years following the diagnosis, with a peak incidence of vertebral fractures at approximately 12 months (3, 5, 6). In addition, different authors have observed reduced bone mineral density (BMD) and a 6-fold increased risk of fragility fractures in long-term survivors of childhood ALL compared to healthy controls (7–10).

Several leukemia-related determinants play a detrimental role in bone health, including cranial radiation therapy (11), glucocorticoids, antimetabolites (e.g., methotrexate) (6, 12–16), reduced weight-bearing activity (17), malnutrition (18), and endocrinopathies (19–21). In 2021, an international panel of 36 experts within the Late Effects of Childhood Cancer Guideline Harmonization Group developed systematic recommendations about surveillance protocols among survivors, highlighting the pivotal detrimental role of cranial/craniospinal and total body irradiation in bone mineralization attainment (22).

Given the crucial role of bone accrual during adolescence, the effects of the above-mentioned factors may be even more deleterious in achieving peak bone mass among CAYA (23).

Along with bone impairment, sarcopenic obesity has been reported as another major late effect (24). Increased fat mass and reduced lean mass tend to occur early following treatment administration, due to changes in energy intake and consumption, inflammation, impaired absorption, and loss of nutrients (18, 25). Reduced physical activity and psychological issues may also have a role in over- or undernutrition (17). Interestingly, changes in body composition (BC) are also observed several years after the end of treatments for childhood ALL (18). A recent study by Marriot and colleagues observed a 43% prevalence of sarcopenic obesity, as long as 10 years or more following ALL diagnosis (24). Nevertheless, evidence about long-term changes in BC among survivors is still limited, as the results are difficult to harmonize due to the absence of standardized methods to evaluate lean and fat mass in CAYA and the lack of a uniformly accepted definition of pediatric sarcopenia.

In addition, though several studies have assessed bone health and BC at different times from diagnosis to adult age, published data on a systematic assessment of the longitudinal changes in BC as well as sex-, height-, and age-adjusted total body less than head (TBLH) and lumbar spine (LS) BMD from ALL diagnosis onward are lacking.

Nevertheless, a growing wealth of literature supports a strong association between BC and BMD (26). However, the mutual influence of the two variables in children treated for ALL has not yet been explored.

With the aim of filling these gaps in knowledge, we conducted the present prospective analysis and report the longitudinal trendlines of BMD, circulating mineral and bone turnover markers and BC in children and adolescents during and after treatment of ALL. Finally, we sought to explore the potential association between the changes in BC and BMD recorded over the observation period.

## Materials and methods

### Study design

We conducted a prospective, longitudinal study, enrolling children and adolescents treated for ALL according to the Italian Association of Pediatric Hematology and Oncology (AIEOP)–BFM (Berlin-Frankfurt-Münster) ALL 2009 protocol (https://kinderkrebsinfo.de/doi/e200606) between March 2012 and April 2016 at the Pediatric Hematology Department of Fondazione IRCCS San Gerardo dei Tintori Hospital in Monza, Italy.

Briefly, the treatment protocol includes the following phases: induction, consolidation, reinduction and maintenance. Over the first three phases, patients receive intensive chemotherapy schedules and high-dose corticosteroids. During maintenance, on the other hand, patients are treated with oral methotrexate and 6-mercaptopurine, with no or little impact on hematopoiesis and the immune system. The protocol overall covers a 24-month period.

Eligible patients underwent a systematic evaluation of BMD and BC by dual-energy X-ray absorptiometry (DXA) scan and bone and mineral metabolism status by biochemical analyses at the time of diagnosis and every 6 months until 1 year after the end of treatment. Overall, patients underwent a complete densitometric and biochemical assessment through seven timepoints: T_0_ (upon diagnosis), T_1_ (6 months after diagnosis), T_2_ (12 months after diagnosis), T_3_ (18 months after diagnosis), T_4_ [24 months after diagnosis, which corresponded to the end of treatment (EoT)], T_5_ (30 months after diagnosis, 6 months after the EoT), T_6_ (36 months after diagnosis, 12 months after the EoT).

Due to cancer-related complications, some patients were unable to receive one or more scheduled DXA scans, while others had to drop out of the study during the observation period.

### Study series

Patients diagnosed with ALL between 3 and 18 years of age and treated according to the AIEOP–BFM ALL 2009 protocol were considered eligible. Exclusion criteria encompassed relapse of ALL during the observation period; previous hematopoietic stem cell transplantation; exposure to chemo- and/or radiotherapy before ALL diagnosis; co-occurrence of diseases or treatments associated with an increased risk for reduced BMD; high-dose corticosteroids started more than 3 days before T_0_ DXA scan; cranial radiation therapy; and an underlying syndrome.

All the parents/caregivers of the patients involved provided written informed consent to data collection and analysis. The study was approved by the local Ethics Committee (Comitato Etico della Brianza—version 5.0, 16 December 2011, approved on 26 January 2012).

### Data collection and analysis

The following variables were collected prospectively at diagnosis and then every 6 months until 1 year after the EoT:

- anthropometric data, presented as height and weight standard deviation score (SDS);
- laboratory data: serum calcium (Ca) and phosphate (P), total alkaline phosphatase (ALP), bone-specific alkaline phosphatase (BAP), parathyroid hormone (PTH), osteocalcin (OC), carboxyterminal telopeptide of collagen I (β-CTX), 25-hydroxyvitamin D (25-OHD), receptor activator of nuclear factor κB ligand (RANKL), osteoprotegerin (OPG), and urine calcium and creatinine. Samples were gathered at the Pediatric Hematology Department of Fondazione IRCCS San Gerardo dei Tintori Hospital (Monza, Italy) in the setting of dedicated clinical evaluations. Patients were fasted from midnight, and samples were collected at 8:00 am.

Biochemical analyses were carried out either in Monza or at Istituto Auxologico Italiano and University of Milan, Milan, Italy.

Serum and urine Ca and P, ALP and BAP were assayed by photometric measurement (Roche/Hitachi system, Roche Diagnostics, Mannheim, Germany), while PTH, OC, and 25OH-D were assayed by electrochemiluminescence (ECLIA, Elecsys/cobas, Roche Diagnostics, Mannheim, Germany). β-CTX levels were also determined by ECLIA (Roche Modular E170, Roche Diagnostics, Mannheim, Germany). RANKL and OPG were measured by sandwich ELISA Quantikine Assay according to the manufacturer’s protocol (quantitative sandwich ELISA for RANKL and Pikokine™ ELISA for OPG; MyBioSource, San Diego, CA, USA).

All biochemical analyses were performed in a single run to avoid inter-assay errors.

### Bone mineral density and body composition assessment

BMD and BC data were obtained by DXA [Hologic densitometer (Discovery A, S/N 83724) Hologic, Marlborough, MA, USA]: areal BMD of the lumbar spine (L1–L4) and TBLH-BMD, lean mass (LM), and fat mass (FM). Appendicular skeletal muscle mass (ASMM) of upper and lower extremities was obtained as a surrogate measure of skeletal muscle mass. TBLH-BMD was preferred to total body BMD due to the changes in the contribution of head to bone mineral content in the growing child and adolescent (27, 28) and was height-adjusted by the DXA pediatric software. Lumbar spine bone mineral apparent density (LS-BMAD), which is the transformation of bone area to estimate the volume of each individual vertebra (L1–L4) (29), was measured with the calculator available at *https://courses.washington.edu/bonephys/opBMAD.html* in order to reduce the confounding influence of short stature (29). DXA measurements were expressed as Z-scores in standard deviations (SD), adjusted for age and sex (30). All the patients enrolled were assessed by a single DXA device over the observation period. All the scans were reviewed by a single expert in the field from the Bone Metabolism Diseases Unit of Istituto Auxologico Italiano, for final harmonization of results.

Height-adjusted ASMM index (ASMI) was calculated as ASMM (kg)/height squared (m^2^). Similarly, fat mass index (FMI) was calculated by dividing FM by body surface (kg/m^2^). To assess BC at different ages, ASMI and FMI were expressed as SDS with reference to age- and sex-specific published values (31).

According to the International Society for Clinical Densitometry (ISCD):

– reduced BMD in children and adolescents has been defined as BMD Z-scores that are less than or equal to −2.0, adjusted for age, sex, and body size as appropriate.
– pediatric osteoporosis is defined by 1) the finding of one or more atraumatic vertebral compression fractures or 2) the combination of a BMD Z-score ≤ −2.0 and a clinically significant fracture history (two or more long-bone fractures before the age of 10 years or three or more long bone fractures at any age up to 19 years) (32).

In adults, sarcopenia has been defined as an ASMI <7.0 kg/m^2^ in men and <5.4 kg/m^2^ in women, along with reduced skeletal muscle strength and/or performance (33). There is no consensus on the definition of pediatric sarcopenia; in addition, there are no standardized methods to diagnose sarcopenia in children (34). In our cohort, low skeletal muscle mass was defined as the finding of ASMI <−2 SDS compared to age- and sex-specific reference ranges (31).

All DXA scans were performed with standard manufacturer-recommended acquisition and analysis protocols. Daily quality control was performed using the manufacturer-supplied calibration phantom to ensure instrument stability. Scans were acquired and analyzed by certified technologists following standardized positioning protocols.

### Fractures assessment

Enrolled children were investigated retrospectively for symptomatic fractures during or after the study period. Unfortunately, vertebral morphometric deformities could not be evaluated systematically.

### Statistical analysis

Descriptive statistics were reported as absolute frequencies and percentages or median and quartiles (Q1, Q3), for categorical and continuous variables, respectively. The correlation between the biochemical analyses and BMD was calculated using the Pearson correlation coefficient. Comparisons between baseline and each follow-up assessment were performed using paired Wilcoxon signed-rank tests, accounting for the matched design. Missing follow-up assessments were not imputed. Each comparison was based on all available paired observations, including only participants with measurements available at both baseline and the corresponding follow-up timepoint; therefore, participants with incomplete follow-up contributed to the analyses whenever paired data were available. The Holm method was considered to adjust for multiple testing, and significance tests were reported in terms of adjusted *p*-values.

In addition, longitudinal changes in LS-BMAD and TBLH-BMD Z-scores across the seven scheduled assessments were evaluated using linear mixed-effects models. Timepoint was included as a categorical fixed effect, and a participant-specific random intercept was included to account for within-subject correlation. Models were fitted using all available observations, including participants with incomplete follow-up. Estimated marginal means were calculated at each timepoint, and comparisons between baseline and each follow-up assessment were adjusted for multiple testing using the Holm method.

Both linear and multivariate regression models were used to assess the potential impact of different variables on BMD and BC. Candidate predictors were first evaluated in univariable linear regression models. Variables included in the multivariable models were selected by considering their clinical and biological relevance, the results of the univariable analyses, and the need to preserve model parsimony given the available sample size. Age and sex were considered clinically relevant potential confounders and were retained irrespective of their univariable statistical significance. Body composition measures were included because of their biologically plausible association with BMD. No automated stepwise variable-selection procedure was applied. False discovery rate correction was applied to the exploratory univariable regression analyses.

For each test, the level of significance was set at 0.05. Data management and statistical analyses were performed using the dplyr, tidyr, rstatix, gtsummary, lme4, lmerTest, and emmeans packages. Data visualization was performed using ggplot2 and ggpubr, while correlation matrices were generated using corrplot. All statistical analyses were performed using open-source R software v.4.2.3 (R Foundation for Statistical Computing, Vienna, Austria).

## Results

### Study population

Out of 216 children diagnosed with ALL at Fondazione IRCCS San Gerardo dei Tintori over the observation period, 49 patients fulfilled the enrollment criteria and were included. The flow diagram represented in **Figure 1** summarizes the stepwise selection process.

**Figure 1:**
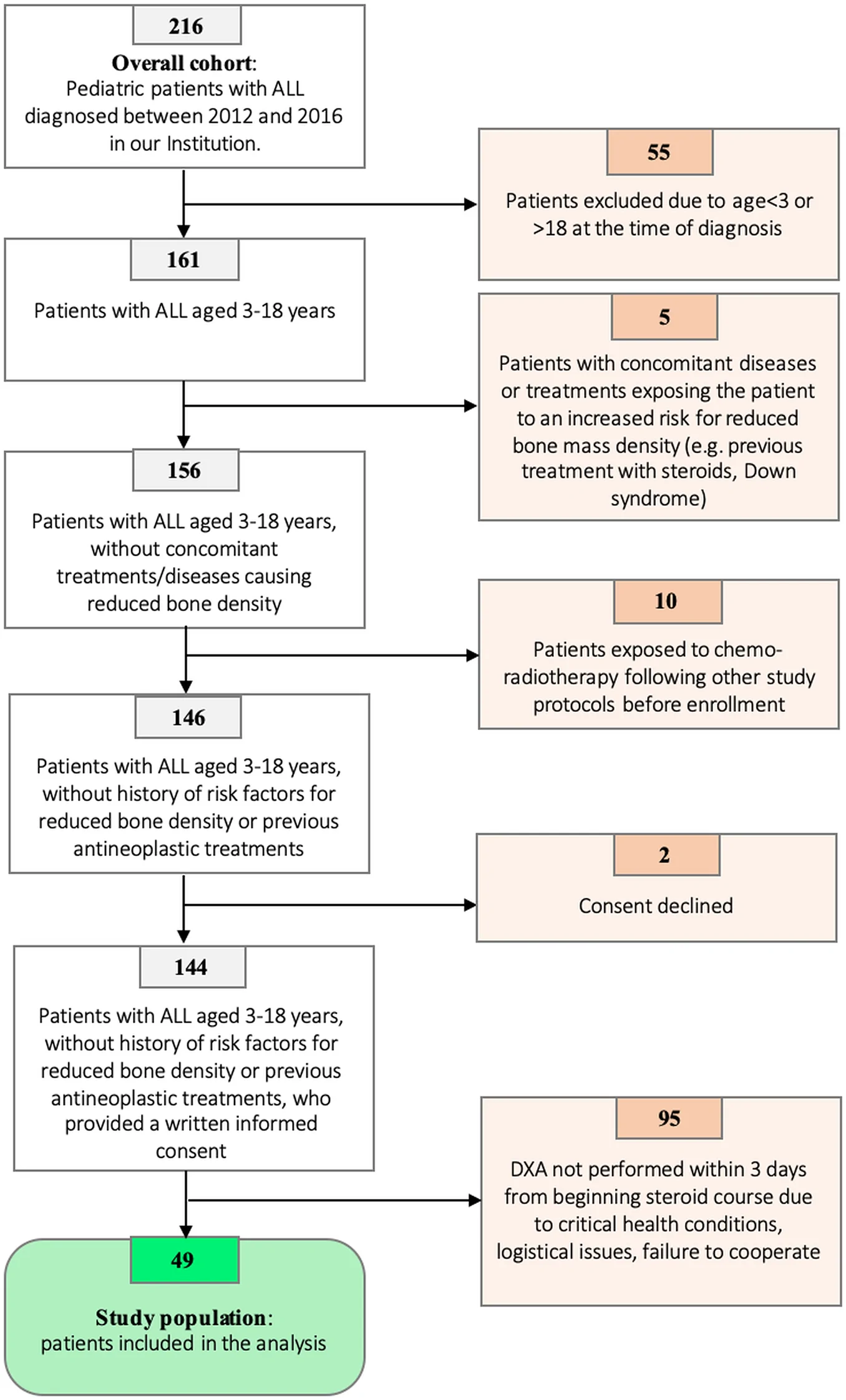
Flow diagram representing the stepwise process that led to the identification of 49 patients enrolled in the present analysis from the 216 diagnosed with acute lymphoblastic leukaemia over the observation period.

Median age at diagnosis was 8.5 years (IQR 6.4–12.6). Forty-one patients (83.7%) were diagnosed with B-cell precursor ALL, the remaining eight (16.3%) with T-cell ALL. Twenty-six patients were female (53.1%).

Of the 49 patients enrolled at T_0_, 31 completed the study and were systematically assessed across all seven timepoints, while the remaining 18 withdrew at various stages between T_1_ and T_6_. Reasons for dropout included i) clinical complications; ii) disease relapse requiring treatment intensification; and iii) withdrawal of informed consent. **Supplementary Table 1** details the number of patients assessed at each timepoint, along with the availability of blood tests and DXA scans from T_0_ to T_6_. **Supplementary Table 2** provides a characterization of the clinical features of the two subcohorts (“completers” and “non-completers”).

### Bone mineral density

All patients enrolled underwent a DXA scan at diagnosis (T_0_). Both lumbar and subtotal BMD were measured in 40/49 patients. Due to technical or clinical issues, LS-BMAD alone was available in six patients, while TBLH alone was assessed in three patients.

LS-BMAD and TBLH-BMD Z-scores at T_0_ were −2.8 [IQR: −3.5, −1.9] and −0.72 [IQR: −1.2, 0.0], respectively. LS-BMAD Z-score was ≤−2.0 in 33 out of the 46 patients for whom it was available (71.7%), while only one subject showed a TBLH-BMD Z-score ≤−2.0 [1/43 patients (2.3%)] (**Table 1**).

**Table 1:** Lumbar spine bone mineral apparent density (LS-BMAD) Z-score, total body less than head bone mineral density (TBLH-BMD) Z-score, lean mass, fat mass, appendicular skeletal muscle mass index (ASMI), and fat mass index (FMI) at diagnosis (T_0_) and 36 months after diagnosis (T_6_).

DXA parameter	T_0_	T_6_	*p-value*	ΔT_2_ − T_0_ (12 months)	ΔT_6_ − T_0_ (36 months)
***LS-BMAD Z-score***	n = 46	n = 31			
median [IQR]	−2.8 [−3.5, −1.9]	−2.0 [−3.0, −0.7]	0.001	−0.2 [−0.5, 0.0]	0.7 [0.2, 1.2]
>−2, n (%)	13 (28.3)	14 (45.2)			
≤−2, n (%)	33 (71.7)	17 (54.8)			
***TBLH-BMD Z score***	n = 43	n = 31			
median [IQR]	−0.7 [−1.2, 0.0]	−1.7 [−2.4, −1.0]	<0.001	−1.5 [−2.3, −1.2]	−0.8 [−1.6, −0.5]
>−2, n (%)	42 (97.7)	18 (58.1)			
≤−2, n (%)	1 (2.3)	13 (41.9)			
***Lean mass, %***	n = 43	n = 31			
median [IQR]	74.7 [71.9, 78.6]	68.7 [62.3, 74.9]	0.0006	−5.7 [−8.2, −3.6]	−5.0 [−9.1, −2.0]
***Fat mass, %***	n = 43	n = 31			
median [IQR]	22.1 [17.9, 25.3]	28.4 [21.9, 35.1]	0.0003	6.1 [3.7, 8.6]	5.21 [1.8, 8.9]
***ASMI-SDS***	n = 43	n = 31			
median [IQR]	0.12 [−0.8, 1.6]	0.03 [−0.98, 0.6]	0.219	−0.6 [−1.2, −0.07]	−0.2 [−0.7,0.33]
***FMI-SDS***	n = 43	n = 31			
median [IQR]	−1.43[−1.9, −1.1]	−0.12 [−1.6, −0.1]	<0.001	0.6 [0.4, 0.9]	0.47 [0.03, 1.1]

As the AIEOP–BFM ALL 2009 protocol is completed at the end of the 24th month of chemotherapy, T_6_ corresponds to 12 months after the end of treatment. ASMI and FMI are reported as SDS with reference to age- and sex-matched published reference ranges (Ofenheimer et al. Reference charts for body composition parameters by dual-energy X-ray absorptiometry in European children and adolescents aged 6 to 18 years—Results from the Austrian LEAD (Lung, hEart, sociAl, boDy) cohort. Pediatr Obes. 2021).

The two columns on the right show the median and the interquartile range (IQR) of the difference calculated for each parameter between T_2_ and T_0_ (span of time elapsed from diagnosis to 12 months after diagnosis, ΔT_2_ − T_0_) and between T_6_ and T_0_ (span of time elapsed from diagnosis to 1 year after the end of therapy, ΔT_6_ − T_0_).

p values <0.05 were reported in bold.

LS-BMAD Z-score values showed a flat trendline over the 24-month treatment phase (from T_0_ to T_4_), followed by a slightly ascending pattern thereafter (**Figures 2A, C**). Accordingly, only median LS-BMAD Z-score values recorded in this late recovery phase (T_5_–T_6_) were statistically different from those collected at T_0_ (*p =* 0.006 and 0.001, respectively). Overall, LS-BMAD was still overtly compromised at T_6_ (Z-score <−2.0 in 54.8% of patients—**Figure 3A**).

**Figure 2:**
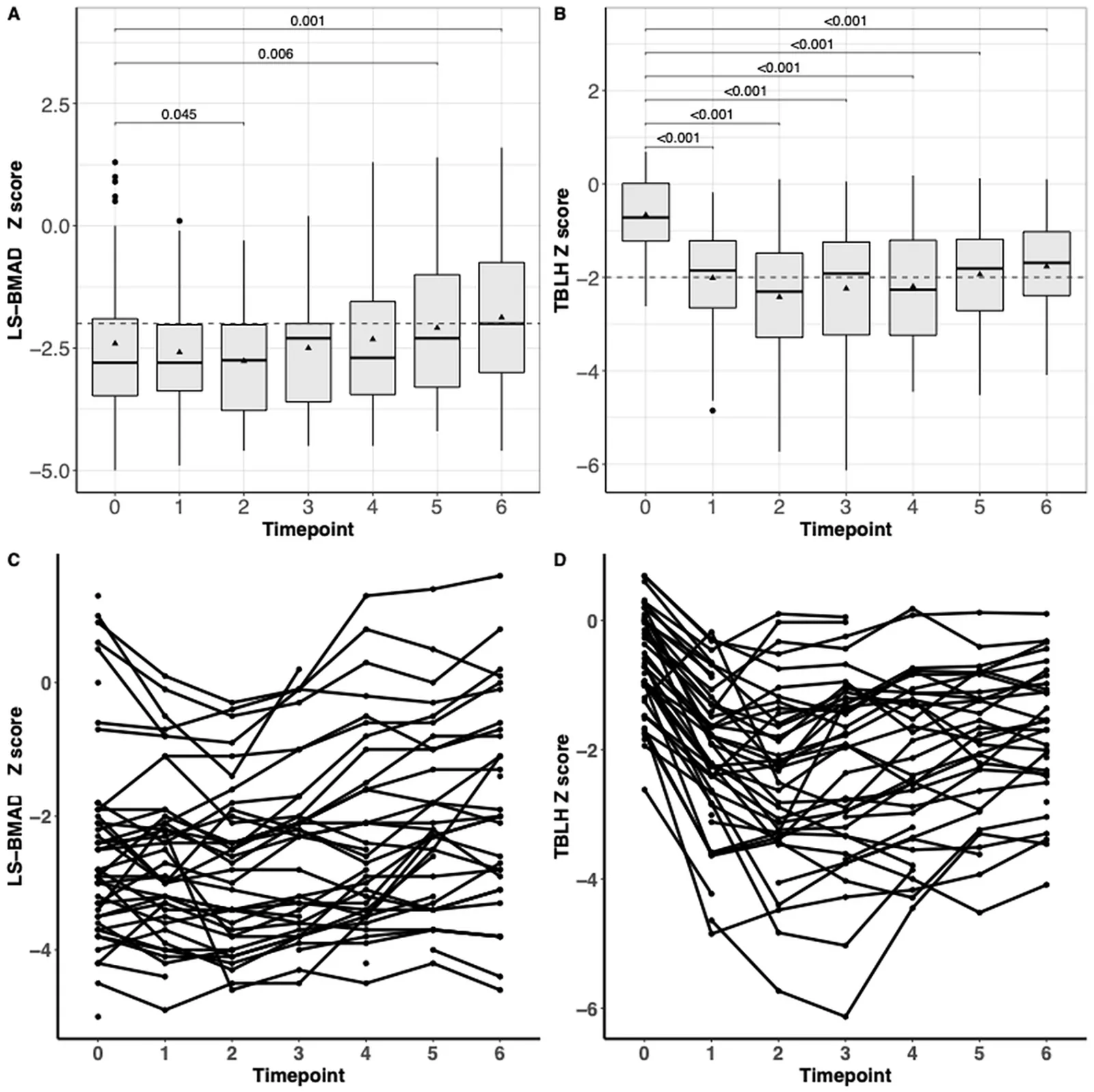
The boxplots (panels **A, B**) show the median and interquartile range of Z-scores of lumbar spine bone mineral apparent density (LS-BMAD Z-score) and the total body less than head bone mineral density (TBLH-BMD Z-score) recorded across the seven sequential timepoints scheduled. The medians of LS-BMAD Z-score and TBLH-BMD Z-score were recorded at diagnosis (T_0_) and then every 6 months until 1 year after the end of treatment (T_6_). The dashed red line represents the cutoff of −2 SDS, used to discriminate between reduced and normal BMD (ISCD criteria). P values of comparisons between BMD at different timepoints and BMD at diagnosis are reported (brackets over the boxplots). Panels **(C, D)** represent the individual evolution of LS-BMAD and TBLH-BMD Z-scores during the follow-up period for each enrolled subject.

**Figure 3:**
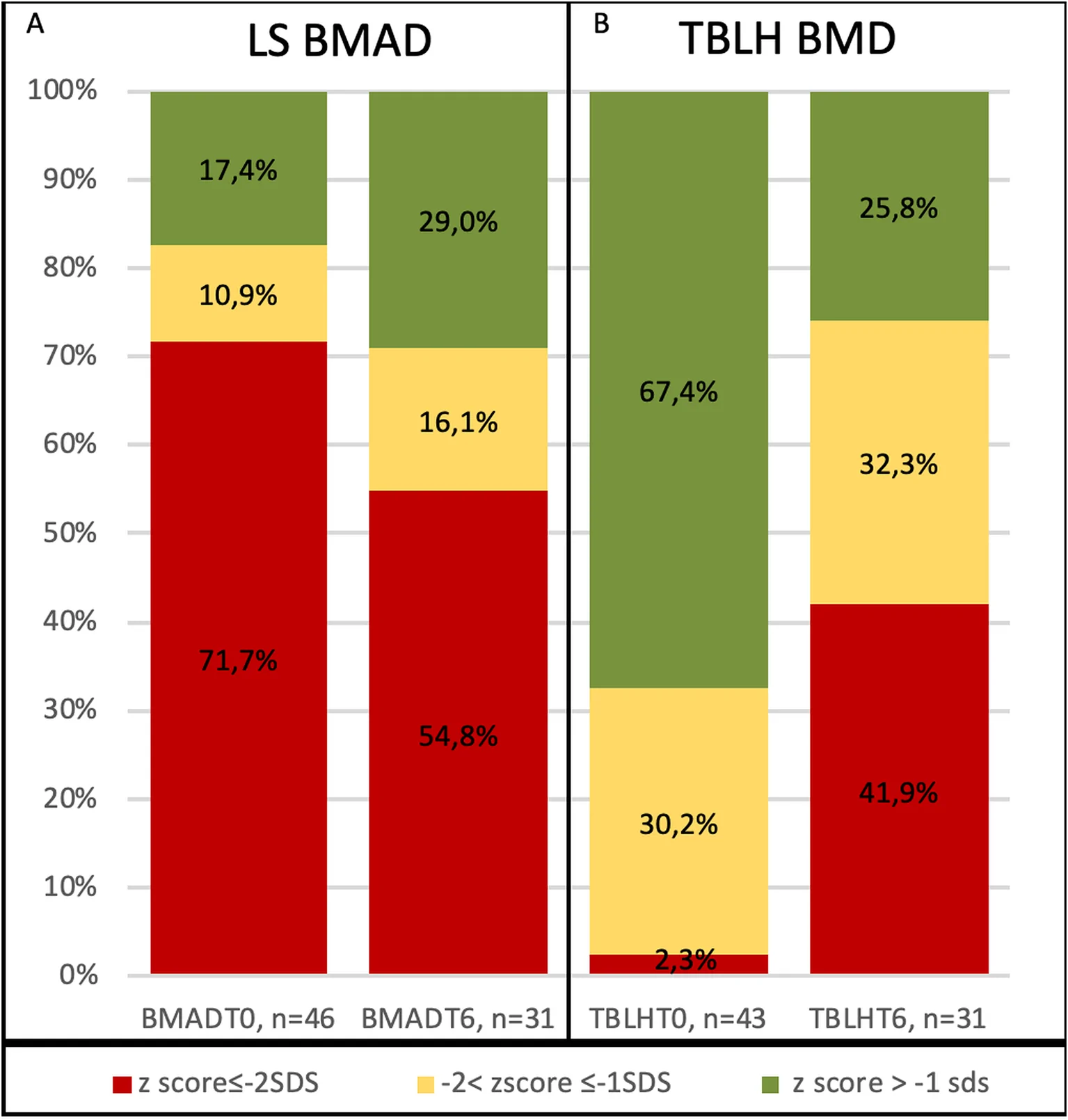
Z-scores of lumbar spine bone mineral apparent density (LS-BMAD Z-score, panel **A**) and total body less than head bone mineral density (TBLH-BMD Z-score, panel **B**) in the study population at diagnosis (T_0_) and 1 year after the end of treatment for leukemia (T_6_). The percentage of children with a Z-score ≤−2 is shown in red, between −2 and less or equal to −1 is illustrated in yellow, and >−1 is shown in green.

Median TBLH-BMD Z-score values showed an overt decline as soon as 6 months after the start of treatment (T_1_), achieving the *nadir* 12 to 18 months following ALL diagnosis (T_2_–T_3_), while a positive trend was recorded from T_3_–T_4_ onward (**Figures 2B, D**). Despite this late-onset improvement, patients did not experience a full recovery of TBLH-BMD during the observation period, and Z-scores recorded at T_6_ were still statistically lower than those recorded at T_0_ (*p* < 0.0001).

At the last timepoint (T_6_), TBLH-BMD Z-scores were ≤−2.0 in 41.9%, >−2.0 and ≤−1.0 in 32.3%, and >−1.0 in 25.8% of patients (**Figure 3B**).

At T_6_, 67.7% of patients showed Z-scores≤−2.0, in LS-BMAD, TBLH-BMD or both.

Of note, children with B-ALL and T-ALL showed statistically superimposable LS-BMAD and TBLH-BMD Z-scores (*p* = 0.891 and 0.852, respectively).

Linear mixed-effects models confirmed a significant overall effect of time on both LS-BMAD [F(6, 208.98) = 14.31, p <0.0001] and TBLH-BMD [F(6, 210.43) = 35.36, p <0.0001] Z-scores. Compared with baseline, estimated LS-BMAD Z-scores were significantly higher at T_5_ (estimated difference: 0.47 SDS, Holm-adjusted p = 0.0003) and T_6_ (estimated difference: 0.62 SDS, Holm-adjusted p <0.0001), while no statistically significant differences were observed at T_1_–T_4_. Conversely, estimated TBLH-BMD Z-scores were significantly lower than baseline at every follow-up assessment, with the largest reduction observed at T_2_ (estimated difference: −1.66 SDS; all Holm-adjusted p-values <0.0001). A progressive but incomplete recovery was subsequently observed, with the estimated TBLH-BMD Z-score at T_6_ remaining 0.96 SDS lower than at baseline.

### Circulating mineral and bone turnover markers

At diagnosis (T_0_), serum Ca and P levels were within the normal range in all patients. Serum 25-OHD levels were below 20 ng/ml in 20 (40.8%) patients, while hyperparathyroidism (plasma PTH >52 pg/ml) (35) was detected in nine (18.4%) patients.

Serum Ca and P concentrations increased after 6 months of therapy, then Ca values remained stable (**Figure 4A**), while serum P levels decreased over time (**Figure 4B**). The trendline for vitamin D is showed in **Figure 4C**. Consistently, the urine calcium-to-urine creatinine *ratio* increased over the first 6 months following diagnosis, then declined over time (**Figure 4D**).

**Figure 4:**
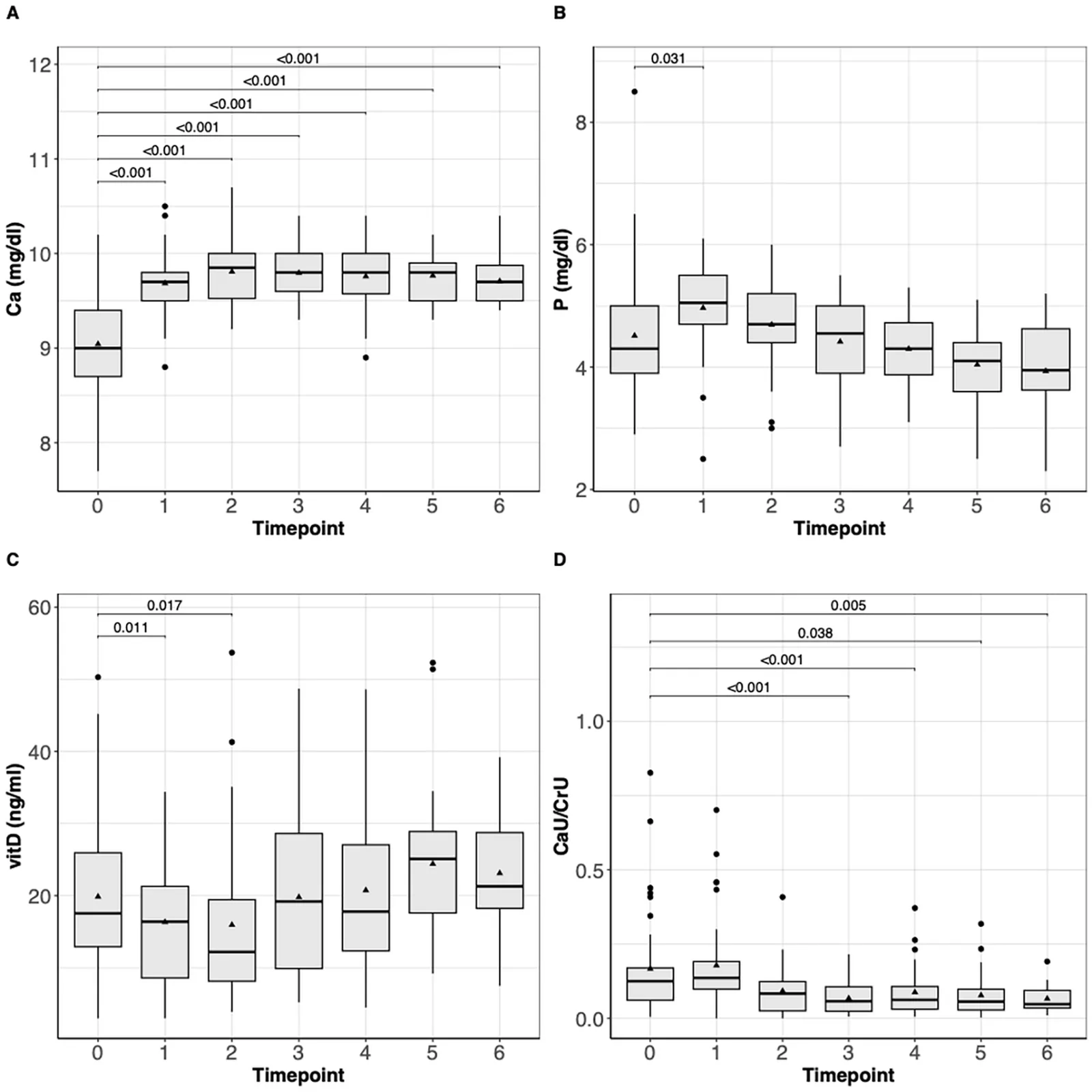
Boxplots reporting the median and interquartile ranges of bone mineral markers: calcium (Ca, panel **A**), phosphate (P, panel **B**), 25-hydroxyvitamin D (25-OHD, panel **C**), and urine calcium/urine creatinine ratio (UCa/UCr, panel **D**) at different timepoints. Laboratory data were recorded at diagnosis (T0) and then every 6 months until 1 year after the end of treatment (T6).

PTH levels showed a progressive increase over the 24-month treatment phase but decreased thereafter. Nevertheless, the difference between PTH levels at any timepoint and T_0_ did not achieve statistical significance (**Figure 5A**).

**Figure 5:**
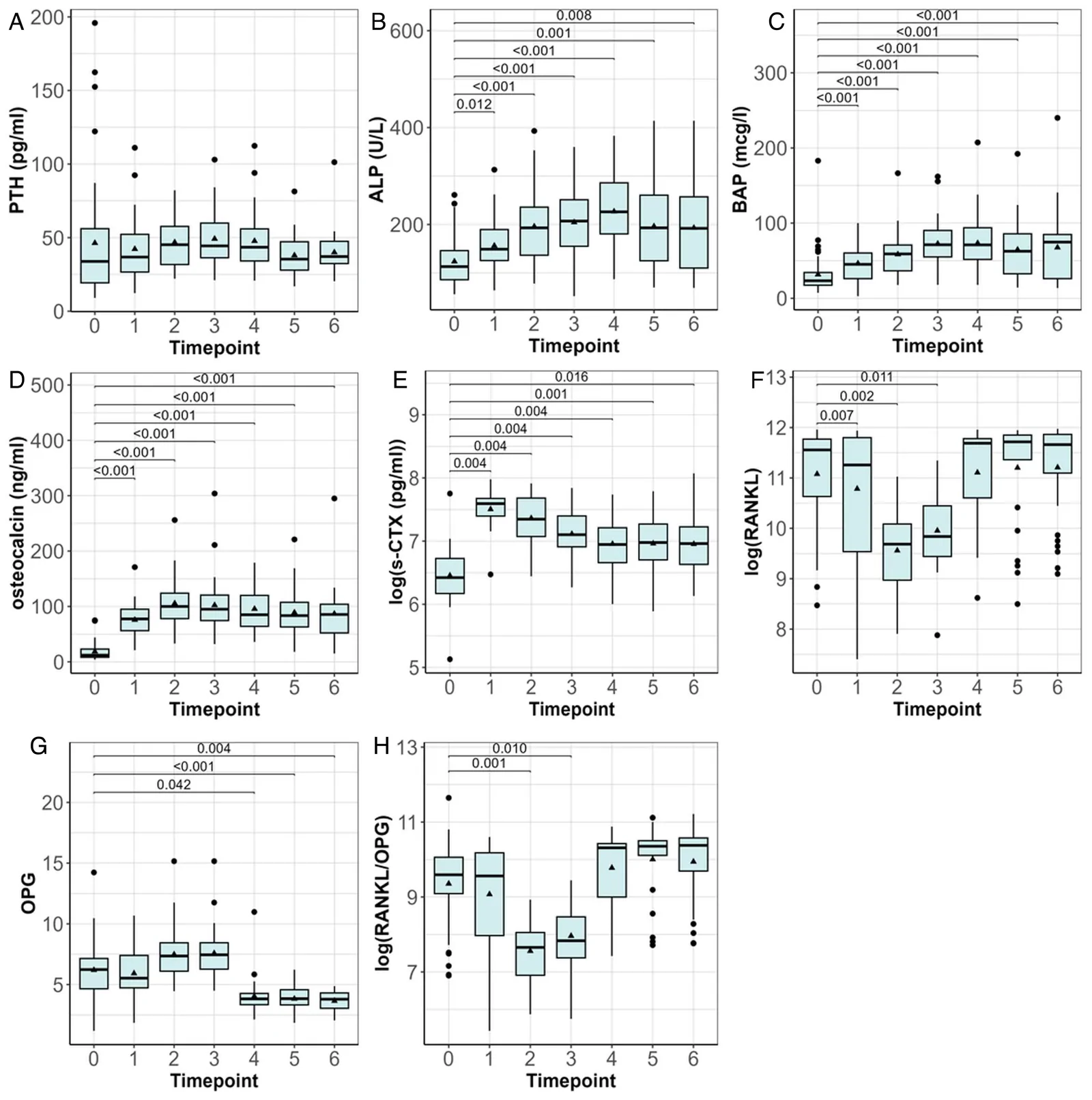
Boxplots reporting the median and interquartile ranges of bone turnover markers: parathyroid hormone (PTH, panel **A**), alkaline phosphatase (ALP, panel **B**), bone-specific alkaline phosphatase (BAP, panel **C**), OC (panel **D**), carboxyterminal telopeptide of collagen 1 (CTX, panel **E**), receptor activator of nuclear factor κ B ligand (RANKL, panel **F**), osteoprotegerin (OPG, panel **G**), and the RANKL/OPG ratio (panel **H**) at different timepoints. Laboratory data were recorded at diagnosis (T_0_) and then every 6 months until 1 year after the end of treatment (T_6_).

Circulating markers of bone formation (i.e., ALP, BAP, and OC) showed a consistent and statistically significant increase over the early treatment phases, with overall stable values during maintenance and after the end of treatment (**Figures 5B–D**).

Focusing on circulating markers of bone resorption, we found a remarkable and prompt increase in β-CTX median values as soon as 6 months after diagnosis, followed by a progressive decline over time (**Figure 5E**). In addition, our data highlighted a specular trend for RANKL and OPG values, with the latter presenting an isolated increase between 12 and 18 months of treatment and the former showing a co-occurrent remarkable decrease at the same timepoints (**Figures 5F–H**).

Univariate analyses showed that none of the circulating markers correlated with LS-BMAD or TBLH-BMD Z-scores at T_0_.

The matrices reported in **Supplementary Figure 1** provide a graphical representation of all the correlation coefficients recorded between pairs of biochemical and/or densitometric parameters at T_0_, T_2_, and T_6_.

Biochemical markers of bone turnover poorly correlated with LS-BMAD and TBLH-BMD Z-scores at any timepoint, except for ALP, which showed a statistically significant negative correlation with LS-BMAD Z-score at T_6_ (r = −0.65, *p* = 0.00016).

### Body composition

The longitudinal assessment of BC highlighted a remarkable and statistically significant decrease in the percentage of LM from T_1_ onward (**Figure 6A**). At T_6_, the median percentage of LM was 68.7% (IQR 62.3, 74.9), still statistically and remarkably lower than at T_0_ [74.7% (IQR 71.9, 78.6), *p* = 0.0006]. In addition, ASMI-SDS levels showed a downward trend at T_1_–T_2_ but recovered subsequently, with no statistically significant differences between T_6_ [0.03 (IQR −0.98, 0.6)] and T_0_ [0.12 (IQR −0.8, 1.6); *p =* 0.219; **Figure 6C**].

**Figure 6:**
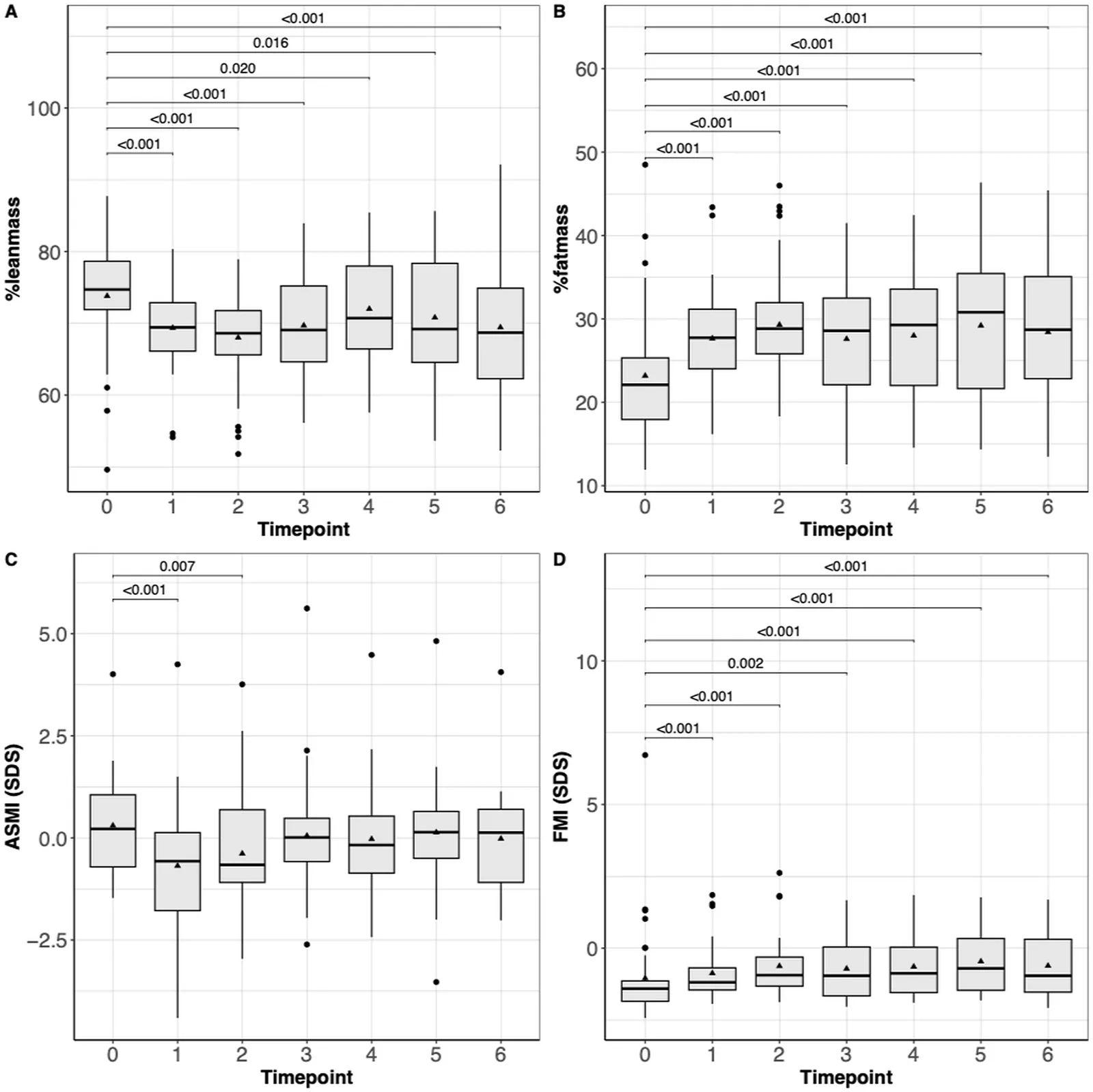
Boxplots showing the longitudinal changes in median and interquartile ranges for lean mass (%, panel **A**), fat mass (%, panel **B**), appendicular skeletal muscle mass index standard deviation score (ASMI-SDS, panel **C**), and fat mass index standard deviation score (FMI-SDS, panel **D**) from T_0_ to T_6_. BC data were recorded at diagnosis (T_0_) and then every 6 months until 1 year after the end of treatment (T_6_). P values of comparisons between BC data at different timepoints and BC data at diagnosis are illustrated (brackets over the boxplots).

Conversely, the percentage of FM progressively increased from T_1_ onward with median values being statistically greater than at T_0_ at every subsequent observation timepoint (*p* < 0.001; **Figure 6B**). Consistently, FMI levels remarkably increased during the treatment phase, and FMI-SDS levels at T_6_ were still greater than at T_0_ [−0.12 (IQR −1.6, −0.1) vs. −1.43 (IQR −1.9, −1.1), *p* < 0.001]. The trendline of FMI-SDS in reported in **Figure 6D**.

The median and IQR for LM (%), FM (%), ASMI-SDS, and FMI-SDS at T_0_ and T_6_ are reported in **Table 1**.

### The impact of demographics, biochemical and hormonal data, and body composition on bone mineral density

LS-BMAD Z-scores at T_0_ were positively affected by age at diagnosis (univariate analysis, *p* < 0.001, **Table 2**). At multivariable assessment, both TBLH-BMD and LS-BMAD Z-scores at T_0_ were positively influenced by age at diagnosis (*p* = 0.027 and <0.001, respectively, **Table 3**). By contrast, age at diagnosis negatively affected the longitudinal improvements in TBLH-BMD over time (ΔTBLH-BMD T_6_–T_0_, *p* = 0.009, **Table 4**).

**Table 2:** Univariate analysis of the factors affecting total body less than head bone mineral density (TBLH-BMD) and lumbar spine bone mineral apparent density (LS-BMAD) Z-scores upon diagnosis (T_0_).

Characteristic	TBLH-BMD Z-score T_0_					LS-BMAD Z-score T_0_				
	N	Beta	95% CI	p-value	q-value	N	Beta	95% CI	p-value	q-value
**Age at diagnosis**	43	0.03	−0.04, 0.09	0.454	0.616	46	0.27	0.18, 0.36	**<0.001**	**<0.001**
**Gender**	43					46				
*F*		–	–				–	–		
*M*		−0.15	−0.65, 0.36	0.562	0.712		−0.48	−1.4, 0.43	0.295	0.547
**Risk group**	43					46				
*HR*		–	–				–	–		
*no HR*		0.54	−0.12, 1.2	0.106	0.410		0.62	−0.47, 1.7	0.261	0.547
**B/T ALL**	43					46				
*ALL B*		–	–				–	–		
*ALL T*		−0.07	−0.72, 0.58	0.837	0.837		−0.08	−1.3, 1.1	0.890	0.890
**Ca T_0_**	43	−0.28	−0.75, 0.18	0.230	0.463	46	−0.52	−1.4, 0.33	0.224	0.531
**P T_0_**	43	0.15	−0.11, 0.41	0.243	0.463	46	−0.37	−0.84, 0.10	0.120	0.327
**CaU/CrU T_0_**	35	−0.37	−2.0, 1.3	0.652	0.768	36	−2.3	−5.3, 0.60	0.115	0.327
**PTH T_0_**	41	0.01	0.00, 0.01	0.129	0.410	45	0.01	0.00, 0.02	0.075	0.286
**Osteocalcin T_0_**	28	0.01	−0.01, 0.03	0.374	0.616	32	0.01	−0.02, 0.03	0.458	0.621
**vitD T_0_**	42	0.02	0.00, 0.04	0.061	0.410	45	−0.01	−0.06, 0.03	0.505	0.639
**ALP T_0_**	43	0.00	0.00, 0.01	0.110	0.410	46	0.00	−0.01, 0.01	0.695	0.733
**BAP T_0_**	42	0.00	−0.01, 0.01	0.725	0.768	45	0.00	−0.02, 0.01	0.600	0.713
**log_CTX T_0_**	14	0.68	−0.37, 1.7	0.182	0.432	13	−0.61	−2.2, 1.0	0.422	0.617
**ASMI-SDS T_0_**	39	0.41	0.22, 0.59	**<0.001**	**0.002**	38	0.20	−0.25, 0.64	0.371	0.587
**FMI-SDS T_0_**	39	0.11	−0.05, 0.28	0.169	0.432	38	0.42	0.12, 0.71	**0.007**	**0.044**
**Log RANKL T_0_**	32	−0.05	−0.34, 0.24	0.728	0.768	34	−0.24	−0.73, 0.24	0.317	0.547
**OPG T_0_**	32	−0.11	−0.24, 0.02	0.099	0.410	34	−0.04	−0.25, 0.17	0.690	0.733

HR, high-risk; ALL, acute lymphoblastic leukemia; Ca, Calcium; P, phosphate; CaU/CrU, urine calcium-to-urine creatinine ratio; PTH, parathyroid hormone; vitD, 25-OHD; ALP, alkaline phosphatase; BAP, bone alkaline phosphatase; CTX, C-terminal telopeptide of type 1 collagen, ASMI, appendicular skeletal muscle mass index; FMI, fat mass index; SDS, standard deviation score; RANKL, receptor Activator of Nuclear Factor κ B ligand; OPG, osteoprotegerin, CI, Confidence Interval, q-value, false discovery rate correction for multiple testing.

p values <0.05 were reported in bold.

**Table 3:** Multivariable analysis of the factors affecting total body less than head bone mineral density (TBLH-BMD) and lumbar spine bone mineral apparent density (LS-BMAD) Z-scores upon diagnosis (T_0_).

Characteristic	TBLH-BMD Z-score			LS-BMAD Z-score		
	Beta	95% CI	p-value	Beta	95% CI	p-value
**Age at diagnosis**	0.07	0.01, 0.14	**0.027**	0.32	0.21, 0.43	**<0.001**
**Gender**			0.840			0.333
*F*	–	–		–	–	
*M*	0.04	−0.38, 0.47		−0.32	−0.99, 0.35	
**ASMI-SDS T_0_**	0.46	0.25, 0.67	**<0.001**	0.10	−0.22, 0.43	0.525
**FMI-SDS T_0_**	−0.08	−0.23, 0.08	0.322	0.15	−0.09, 0.40	0.211

ASMI, appendicular skeletal muscle mass index; FMI, fat mass index; SDS, standard deviation score, CI, confidence interval.

p values <0.05 were reported in bold.

**Table 4:** Multivariable analysis of the factors affecting the longitudinal changes recorded in total body less than head bone mineral density (ΔTBLH-BMD Z-score T_6_ − T_0_) and lumbar spine bone mineral apparent density (ΔLS-BMAD Z-score T_6_ − T_0_) during the study observation period.

Characteristic	Δ TBLH-BMD T_6_ − T_0_			Δ LS-BMAD T_6_ − T_0_		
	Beta	95% CI	p-value	Beta	95% CI	p-value
**Gender**			0.263			0.901
*F*	–	–		–	–	
*M*	−0.35	−1.0, 0.29		0.04	−0.66, 0.75	
**Age at diagnosis**	−0.20	−0.34, −0.06	***0.009***	0.13	−0.02, 0.28	0.091
**B/T ALL**			0.809			0.208
*LLA B*	–	–		–	–	
*LLA T*	−0.09	−0.87, 0.69		−0.53	−1.4, 0.32	
**Δ ASMI-SDS T_6_**− **T_0_**	0.60	0.17, 1.0	***0.008***	0.11	−0.35, 0.57	0.611
**Δ FMI SDS T_6_**− **T_0_**	0.26	−0.20, 0.71	0.249	−0.27	−0.76, 0.23	0.269

The following covariates were included: sex, age at diagnosis, ALL immunophenotype, Δ ASMI-SDS T_6_ − T_0_ and Δ FMI SDS T_6_ − T_0_.

Δ ASMI-SDS T_6_ − T_0_ and Δ FMI SDS T_6_ − T_0_ are referred to the changes in ASMI- and FMI-SDS from T_0_ to T_6_.

ALL, acute lymphoblastic leukemia; ASMI, appendicular skeletal muscle mass index; FMI, fat mass index; SDS, standard deviation score; CI, Confidence Interval.

p values <0.05 were reported in bold.

As in univariate analyses neither leukemia phenotype nor risk group nor any bone mineral marker showed a statistical impact on LS-BMAD or TBLH-BMD Z-scores, these were not included in the multivariable analysis.

Univariate analysis showed that at T_0_, TBLH-BMD Z-score was affected by ASMI-SDS values (**Table 2**); the result was confirmed by multivariate analysis (**Table 3**). Furthermore, when assessing the longitudinal changes in BMD over the observation period, a more favorable course in ASMI-SDS values from T_0_ to T_6_ (namely, a higher ΔASMI-SDS T_6_–T_0_) showed a statistically significant positive impact on TBLH-BMD Z-score recovery (ΔTBLH-BMD T_6_–T_0_, *p =* 0.008), but not on LS-BMAD Z-score (*p* = 0.611), as reported in **Table 4**.

### Occurrence of fractures

No symptomatic fractures were recorded during the observation period (between T_0_ and T_6_).

All the patients alive on 30 August 2024 (n = 43) were contacted by phone in order to collect data retrospectively about the subsequent occurrence of fractures. The median time elapsed following ALL diagnosis was 11.6 years [IQR: 10.8, 12.0]. Overall, seven patients experienced at least one fracture, and one individual experienced two fractures. Data about self-reported fractures are summarized in **Table 5**.

**Table 5:** Self-reported fractures occurring after the end of study protocol.

Progressive study number	Gender	Age at fracture (years)	Years elapsed after ALL EoT	Bone(s) involved
#10	F	13.6	2.3	Diaphysis of radius
#18	M	20.7	2.7	Third toe
#32	M	17.0	5.4	Diaphysis of radius
#35	M	14.2	6.4	Diaphysis of radius and ulna*
#44	F	22.1	5.7	Proximal epiphysis of radius
#45	F	10.6	1.9	Third hand finger
#47	F	10.7	2.6	Distal epiphysis of radius
		11.0	2.9	Fifth finger of hand

Of note, patient #47 experienced two fractures (2.6 and 2.9 years after the end of treatment, respectively).

Patient #35, who reported one fracture after the end of treatment, underwent hematopoietic stem cell transplantation following front-line protocol. Accordingly, he received a remarkably higher steroid cumulative dose compared to the other patients listed.

ALL, acute lymphoblastic leukemia; EoT, end of treatment; q-value, false discovery rate correction for multiple testing.

## Discussion

Bone health disorders represent an important *sequela* experienced by CAYA ALL survivors. Reduced BMD and increased risk for fractures have been reported extensively at diagnosis, over the treatment phase, or even several years following completion of therapy (3, 4, 6, 36, 37).

Previously published literature mostly reported data about bone health in adult ALL patients, evaluated by cross-sectional assessments and focusing on LS alone (3, 6, 8, 38).

### Bone mineral density

Our longitudinal data highlight that LS-BMD was already remarkably affected at diagnosis of ALL, with median LS-BMAD Z-score as low as −2.8 before the beginning of chemotherapy. By contrast, TBLH-BMD Z-score at diagnosis was above −2.0 in all but one patient enrolled. A noteworthy reduction in LS-BMAD Z-score at ALL onset has already been reported (3, 8, 39). It has been speculated that this finding may be the result of the detrimental effect exerted by leukemic blasts on the trabecular bone, which is prevalent in lumbar vertebrae and has a more active metabolism than the cortical counterpart. Both malignant cell infiltration and tumor-released cytokines and autocrine factors may potentially affect trabecular bone health, though dedicated studies are warranted to confirm this pathogenic hypothesis (40, 41). In addition, leukemic infiltration mostly involves trabecular rather than cortical bone, as the former is an active site of hematopoiesis and bone marrow plays a non-negligible role in the mechanical properties of trabecular bone in different loading conditions.

Consistent with the strict enrollment criteria of the present analysis, patients with pre-existing clinical conditions potentially affecting bone health and LS-BMAD Z-score had been excluded. Accordingly, confounding factors, i.e., previous corticosteroid exposure, prolonged immobilization, and chronic inflammation, have been ruled out, leading us to speculate about the key pathogenic role of leukemic cells.

Conversely, despite normal average findings at diagnosis, TBLH-BMD Z-score values showed an abrupt decline as soon as 6–12 months after the start of the treatment protocol, owing to the delayed susceptibility of the cortical bone to chemotherapy, glucocorticosteroids, and long-standing immobilization.

The detrimental impact of glucocorticosteroids and high-dose methotrexate on bone health is well documented (14). According to the AIEOP–BFM ALL 2009 protocol, most patients receive intensive treatment within the first year of treatment, which corresponds to the span of time during which the decrease in TBLH-BMD became overt. Conversely, median LS-BMAD Z-score values showed an overall flattened course over the first 24 months after diagnosis, suggesting that anti-leukemic therapy does not play an additional detrimental role in a previously severely compromised vertebral BMD. The literature reports a peak of occurrence of vertebral crushes approximately 12 months after ALL diagnosis (6). We hypothesize that, following the first year of prolonged hospitalization during the most intense treatment phase, children are exposed to an increasingly active lifestyle, potentially leading to a greater occurrence of post-traumatic fractures in the setting of extensively demineralized bones, despite overall stable LS-BMAD (6).

Finally, both TBLH-BMD and LS-BMAD median Z-score levels began to show a progressive recovery from 24 months after diagnosis onward. Nevertheless, as late as 1 year after the end of therapy, TBLH-BMD was far from retrieving baseline Z-scores. Accordingly, though exceeding the severely impaired median values recorded at T_0_, LS-BMAD Z-score median values were still remarkably lower than the reference range for the healthy pediatric population at T_6_.

The trendlines of LS-BMAD over the first 3 years from diagnosis recorded in our study were superimposable on those reported by Winkel et al. (3). Conversely, van der Sluis et al. described a decline in median LS-BMAD Z-score at 8 months without any late-onset recovery (8). Interestingly, BMAD and LS-BMD Z-scores reported by the two above-mentioned studies were higher than those retrieved in our population (3, 8). We speculate that the lack of adjustment for patients’ height and the different therapeutic burden of the treatment protocols assessed (Dutch Childhood Oncology Group (DCOG) - acute lymphoblastic leukaemia-9 (ALL9) protocol versus AIEOP-BFM ALL 2009) may account for the discrepancies.

To the best of our knowledge, no other studies have assessed subtotal body (TBLH)-BMD longitudinal changes over time in children with ALL. Van der Sluis and colleagues outlined a progressive decline in total body BMD over the first 6 to 12 months from diagnosis. Nevertheless, while we documented an initial recovery trend from the end of treatment onward, these authors report an additional decrease in Z-scores thereafter (8). As growth attainment is often severely impaired during ALL treatment, the lack of adjustment for height may result in an overestimation of the decrease in BMD recorded longitudinally. In addition, as BC changes over childhood and adolescence, the inclusion of the skull in the DXA assessment of the pediatric population enrolled by van der Sluis and colleagues may provide an explanation for these apparently conflicting outcomes.

Consistent with previously reported results (3), younger age was found to be a risk factor for a more compromised LS-BMAD at diagnosis. On the other hand, younger children experienced a remarkably milder reduction in TBLH-BMD over time. It could be hypothesized that younger age reflects a greater bone turnover reserve, leading to a more timely and effective BMD recovery.

### Bone mineral and bone turnover markers

At the time of ALL diagnosis, both mineral and bone turnover markers did not show significant alterations. In detail, neither hypercalcemia nor an increase in bone resorption markers was detected. Consistent with previous studies, 25-OHD decreased during the first most intensive phase of treatment, likely due to reduced sunlight exposure and altered dietary intake (42).

Glucocorticosteroids (GCs) were extensively administered during the first 6–8 months of the AIEOP–BFM ALL 2009 protocol. GCs are known to induce osteoclast and osteoblast apoptosis (43). Unexpectedly, after 6 months of treatment, both bone formation and bone resorption markers were increased, suggesting a condition of elevated bone turnover, which was associated with increased serum and urine calcium levels and a compensatory over-activation of parathyroid function. In line with increased bone turnover, serum ALP showed a statistically significant negative correlation with LS-BMAD Z-score as late as 1 year after the end of treatment.

By timepoint T_2_, as corticosteroids had been discontinued for several weeks for all the patients enrolled, bone formation markers started to show a remarkable upward trend.

In addition, the analysis of the RANKL/OPG ratio provided interesting insights. RANKL prompts osteoclast development and survival, promoting bone resorption. Conversely, OPG, a soluble receptor secreted by osteoblasts, binds RANKL and prevents the RANK–RANKL interaction, inhibiting osteoclasts’ activity (44). Accordingly, the RANKL/OPG *ratio* controls the physiological balance between bone formation and resorption, with a higher *ratio* mirroring more vigorous bone resorption phenomena. GCs have been reported to be potent inhibitors of OPG production and also stimulate RANKL. Conversely, methotrexate inhibits osteoclastogenesis by downregulating RANKL secretion (45). In our series, we reported a reduction in the RANKL/OPG *ratio* between T_2_ and T_4_. In light of published literature, we speculate that this finding could be regarded as the synergistic result of the discontinuation of high-dose steroids administered over the first months of treatment and the start of the long-term maintenance treatment phase encompassing oral methotrexate. Maintenance starts at the end of the first year of treatment and lasts until the 24th month after ALL diagnosis. Following the end of treatment (T_4_), bone turnover activity showed an upward trend, as demonstrated by the normalized RANKL/OPG *ratio*.

### Body composition

With reference to BC, we found that indexes of appendicular lean mass (namely, the ASMI-SDS) correlated statistically with TBLH-BMD Z-score at diagnosis, confirming the described association between BC and BMD (26).

In addition, the magnitude of the improvements recorded longitudinally in ASMI-SDS was statistically associated with the gain in TBLH-BMD Z-score at T_6_. As an increasing wealth of published literature corroborates the mechanistic relationship between LM and BMD (26), these findings led us to speculate that prolonged immobilization and decline in lean body mass may represent key determinants of bone mineral loss among ALL survivors, although further dedicated analyses are warranted to confirm this hypothesis. If confirmed, this conclusion would suggest that the timely promotion of weight-bearing exercise could prevent children from experiencing both LM and cortical BMD decline following ALL treatment.

While LM showed a progressively declining trendline, FM persistently increased over the observation period, consistent with the reports from other populations of CAYA survivors (8, 18). While cross-sectional studies have assessed the prevalence of sarcopenic obesity as late as 10 years after treatment for childhood ALL (24), ours is the first analysis reporting longitudinal trends of LM, FM, and ASMI/FMI over time. In addition, one of the strengths of the present assessment is the fact that all the ASMI and FMI indexes have been normalized according to age- and sex-related pediatric references and have been accordingly expressed as SDS. This systematic approach provides our analysis with remarkable formal accuracy, as the standardized cut-offs for sarcopenia and sarcopenic obesity used to assess BC derangements in adults with cancer cannot be transferred to pediatrics *tout-court*. In addition, the use of ASMI and FMI as absolute values in the setting of a longitudinal assessment of BC would have resulted in an over- or underestimation of the magnitude of abnormal findings, as these indexes change physiologically in the growing body of children and adolescents.

The present study admittedly suffers from some limitations: 1) limited sample size; 2) lack of systematic assessment of vertebral morphometry; 3) not all the patients enrolled could undergo all seven DXA scans from T_0_ to T_6_; and 4) the study includes a wide span of age for the patients enrolled, ranging from childhood to late adolescence. Pubertal development could have played a role in BMD and biochemical marker changes over time; nevertheless, assessing the relative contribution of sex hormones to these variables would be extremely difficult.

Concerning spine morphometry, asymptomatic vertebral crushes are well documented in children with ALL, occurring in up to approximately one-quarter of patients at or around diagnosis (46), and their detection requires dedicated spinal radiography or vertebral fracture assessment (VFA), which was not performed in our cohort. This choice was deliberate: systematic spinal imaging at each of the seven timepoints would have imposed a significant cumulative radiation burden in a pediatric population, and no current international guideline (including the Children’s Oncology Group Long-Term Follow-Up Guidelines) recommends universal radiographic spinal screening in the absence of clinical symptoms (47). Furthermore, the natural history of vertebral fractures in this population is frequently characterized by spontaneous remodelling following antineoplastic treatment, limiting the therapeutic implications of their systematic detection in asymptomatic patients (48). Future studies integrating low-dose VFA technology alongside DXA—thus minimizing radiation exposure—would be valuable to comprehensively characterize musculoskeletal outcomes in this population.

Finally, during the enrollment phase, 95 patients could not be included as a DXA scan could not be performed within the first 72 h following ALL diagnosis. We acknowledge that this could be regarded as a potential source of selection bias. Nevertheless, we believe that this concern should be interpreted with caution, as the vast majority of these exclusions were caused by purely logistical constraints rather than by clinical decision-making related to disease severity. In our institution, DXA scans are not available daily, while corticosteroid therapy must be initiated as soon as the diagnosis is established, irrespective of the scheduled DXA availability. Only a few patients were excluded due to the need for more urgent steroid initiation or severe clinical presentation. Accordingly, it is unlikely that these exclusions could have substantially skewed our final cohort. Nonetheless, we recognize this limitation and encourage future multicenter studies to confirm the generalizability of our findings across the full spectrum of clinical presentations at leukemia onset.

In addition, the study strengths include 1) prospective study design; 2) the 49 patients underwent over 250 DXA scans; 3) the observation period extended throughout 36 months; and 4) strict inclusion criteria and study design, aiming to achieve high-quality and reproducible data. For example, all the patients enrolled had a T_0_ DXA scan performed within 72 h after the start of glucocorticosteroids. As a consequence, DXA could not be performed immediately in severely ill patients, who had to be excluded from the analyses. Consistently, in case of severe co-occurring treatment- or disease-related complications, follow-up DXA scans could be performed beyond the strict timetable scheduled for each timepoint and therefore be labelled as missing. Overall, the rarity of the disease, the strict formal follow-up protocol, and the prospective study design shed light on the quality of the assessment performed.

## Conclusions

In conclusion, our results highlight that, among children treated for ALL, LS-BMAD is remarkably affected at diagnosis, while TBLH-BMD, mostly normal at T_0_, declines promptly as soon as 6–12 months after the start of treatment. Both LS-BMAD and TBLH-BMD show an improving trend after the discontinuation of chemotherapy, but 3 years after diagnosis either LS-BMAD or TBLH-BMD Z-score ≤−2.0 is still a prevalent finding among CAYA ALL survivors. The BMD changes are associated with increased bone turnover and parathyroid function activation.

In addition, children and adolescents experience a decline in lean body mass and a co-occurrent increase in fat mass over ALL treatment. The recovery trendlines of appendicular lean mass are statistically associated with the longitudinal improvements of subtotal BMD, suggesting that preventing children and adolescents from experiencing LM loss over time may be regarded as a protective determinant of total body BMD as late as 3 years after ALL diagnosis.

## Data Availability

The raw data supporting the conclusions of this article will be made available by the authors, without undue reservation.

## Supplementary material

Supplementary Figure 1 Matrix of the correlation coefficients between couples of biochemical variables. Panels A, C and E report the Pearson’s coefficients for all the biochemical variables plotted against Z score of lumbar spine bone mineral apparent density at T_0_, T_2_ and T_6_, respectively. Panels B, D and F report the coefficient of correlation with reference to total body less than head bone mineral density Z-score at T_0_, T_2_ and T_6_, respectively. The greater the coefficient, the more intense is the color of each number, with red representing negative correlations and blue positive ones. ALP – alkaline phosphatase; BAP – bone-specific alkaline phosphatase; BMAD - bone mineral apparent density; BMD - bone mineral density; Ca – calcium; P – phosphate; CaU/CrU– urinary calcium/urinary creatinine ratio; CTX – C-terminal telopeptide of type 1 collagen, OPG - osteoprotegerin; PTH – parathyroid hormone; vitD – 25OH vitamin D.
