# Supplementary material for: Longitudinal changes in bone mineral density, bone turnover markers, and body composition in children and adolescents treated for acute lymphoblastic leukemia
Source: Front Endocrinol (Lausanne). 2026 Oct 19;17:1901073. doi: 10.3389/fendo.2026.1901073 (PMC13724381; doi:10.3389/fendo.2026.1901073)
Supplement: Supplementary file 1 [file Table1.docx]

**Supplementary Table 1**

| Timepoint | Patients permanently dropped out from the study | | | Patients enrolled at each timepoint | Patients still enrolled but DEXA not performed | | | TBLH DEXA available for each timepoint | LS-BMAD available for each timepoint | Blood samples available at each timepoint |
| --- | --- | --- | --- | --- | --- | --- | --- | --- | --- | --- |
|  | For disease relapse/ progression | For persistent complications | Consent withdrawn |  | Both TBLH and LS Scan not performed for co-occurrent complications | TBLH DEXA not performed for logistic/technical issues | LS DEXA not performed for logistic/technical issues |  |  |  |
| T0 | - | - | - | 49 | 0 | 6 | 3 | 43 | 46 | 49 |
| T1 | 0 | 4 | 1 | 44 | 2 | 2 | 0 | 40 | 42 | 44 |
| T2 | 1 | 2 | 0 | 41 | 2 | 3 | 0 | 36 | 39 | 41 |
| T3 | 0 | 1 | 0 | 40 | 0 | 0 | 0 | 40 | 40 | 40 |
| T4 | 1 | 2 | 0 | 37 | 0 | 0 | 0 | 37 | 37 | 37 |
| T5 | 3 | 0 | 0 | 34 | 2 | 0 | 0 | 32 | 32 | 34 |
| T6 | 3 | 0 | 0 | 31 | 0 | 0 | 0 | 31 | 31 | 31 |

**Supplementary Table 1.** Number of patients enrolled at each time point, along with the reasons of drop out and the number of DEXA scans and blood tests available.
