# Supplementary material for: Longitudinal changes in bone mineral density, bone turnover markers, and body composition in children and adolescents treated for acute lymphoblastic leukemia
Source: Front Endocrinol (Lausanne). 2026 Oct 19;17:1901073. doi: 10.3389/fendo.2026.1901073 (PMC13724381; doi:10.3389/fendo.2026.1901073)
Supplement: Supplementary file 2 [file Table2.docx]

**Supplementary Table 2**

|  | | Completers | Non- completers | p-value |
| --- | --- | --- | --- | --- |
| Number of patients | | 31 | 18 | - |
| Age at diagnosis | | 9.9±3.5 | 9.2±4.6 | 0.55 |
| Sex | M | 16 | 7 | 0.56 |
|  | F | 15 | 11 |  |
| Leukemic cell features | B cell ALL | 25 | 16 | 0.69 |
|  | T cell ALL | 6 | 2 |  |
| Disease risk class | Standard risk | 6 | 6 | 0.13 |
|  | Medium risk | 16 | 4 |  |
|  | High risk | 9 | 8 |  |
| SNC involvement at diagnosis | No | 28 | 18 | 0.29 |
|  | Yes | 3 | 0 |  |

**Supplementary table 2**. Characteristics of the 31 patients assessed at every timepoint and of the 18 patients who dropped out over the observation period of the study.

No statistically significant differences were found in terms of clinical and demographic features.
